# Alterations in the IGF-System and Antioxidant Biomarkers in Young Brazilian Adults with Type 1 Diabetes: An Analysis of Cardiovascular Risk Factors

**DOI:** 10.3390/antiox14121514

**Published:** 2025-12-17

**Authors:** Michael Tekle, Diane Meyre Rassi, Eduardo Antonio Donadi, Jacob Grunler, Gustav Dallner, Elisabete Forsberg, Kerstin Brismar

**Affiliations:** 1 Rolf Luft Research Center for Diabetes and Endocrinology, Department of Molecular Medicine and Surgery, Karolinska Institutet, SE-17177 Stockholm, Sweden; 2 Department of Clinical Pharmacology, Karolinska University Hospital, SE-17177 Stockholm, Sweden; 3 Hospital das Clínicas de Ribeirão Preto, Universidade de São Paulo, Avenida Bandeirantes, 3900, Ribeirão Preto 14049-900, Brazil

**Keywords:** type 1 diabetes mellitus, oxidative stress, coenzyme Q10, insulin-like growth factor-I, insulin-like growth factor binding protein-1, glutaredoxin, oxidized low-density lipoprotein

## Abstract

Chronic hyperglycemia inflicts serious cellular damage by inducing oxidative stress through the excessive production of free radicals. This oxidative milieu may impair the cellular redox capacity and disrupt the insulin-like growth factor (IGF) system, thereby increasing the risk of cardiovascular complications. This study aimed to investigate plasma levels of components of the IGF system and antioxidant biomarkers in young adults with type 1 diabetes mellitus (T1DM) compared to age-matched healthy controls in Brazil. This study included 129 patients with T1DM (76 female, 53 male; mean age 26.97 ± 0.6 years) and 95 healthy controls (61 female, 34 male; mean age 27.35 ± 0.68 years). Young Brazilian adults with T1DM had significantly lower mean IGF-I and higher mean IGFBP-1 levels compared to healthy controls. The T1DM group showed a more atherogenic profile, characterized by a significantly elevated ApoB/ApoA1 ratio and increased oxidized LDL levels. However, a subset of patients with significantly better glycemic control exhibited serum IGF-I and IGFBP-1 levels within the normal range observed in controls, which may indicate the presence of residual functional beta-cell activity or reflect better glycemic control in this subgroup. Antioxidant components and oxidative stress biomarkers were significantly upregulated in the T1DM group compared to the control group, suggesting a compensatory adaptive response. No significant correlation was observed between biomarkers of oxidative stress and the IGF-system.

## 1. Introduction

In diabetes, chronic hyperglycemia leads to increased production of reactive oxygen species (ROS) and impairment of the endogenous antioxidant defense system, resulting in elevated oxidative stress [1,2]. Persistently high levels of blood glucose and free fatty acids are associated with mitochondrial ROS overproduction due to redox imbalance [3,4,5]. This contributes to increased oxidation of low-density lipoprotein (LDL), which plays a key role in the initiation and progression of atherosclerosis and other diabetes-related complications [6]. Furthermore, beyond the oxidation of individual lipids, the overall balance of pro- and anti-atherogenic lipoproteins is crucial. The apolipoprotein B to apolipoprotein A1 (ApoB/ApoA1) ratio is increasingly recognized as a potent, integrated marker of cardiovascular risk. ApoB reflects the total number of atherogenic particles, including LDL, while ApoA1 is the main component of anti-atherogenic HDL. Therefore, this ratio was determined in the present study to provide a comprehensive assessment of the cardiovascular risk profile [7].

Cells possess enzymatic and non-enzymatic antioxidant systems, along with complex stress-response networks that help mitigate the effects of free radicals [8]. In the early stages of diabetes, these defense systems are typically upregulated as an adaptive response to environmental stressors [9]. Key antioxidants include coenzyme Q10 (CoQ10) and vitamin E, which are lipid-soluble molecules present in all cell membranes. Endogenously synthesized CoQ10 is a component of the mitochondrial respiratory chain and serves various cellular functions, including acting as an antioxidant [10], whereas vitamin E must be obtained through the diet [11]. Glutaredoxin (Grx) is a small, water-soluble redox enzyme found in both the cytosol and mitochondria, where it participates in critical redox reactions [12,13]. We previously showed that reduced Grx activity was associated with elevated oxidized LDL (ox-LDL) levels, implying a link between Grx activity and oxidative stress [14].

Both oxidative stress and dysregulation of the insulin-like growth factor (IGF) system are believed to contribute to the development of late-stage cardiovascular complications in type 1 diabetes mellitus (T1DM). The IGF system is critical for regulating cellular growth, proliferation, and differentiation, and its disruption can result in pathological conditions, including diabetes and cancer [15,16]. Circulating IGF-I, primarily produced in the liver, is a peptide hormone whose activity is regulated by insulin-like growth factor binding proteins, primarily IGFBP-3, which binds circulating IGF-I and regulates its activity at the cellular level. A small free fraction of IGF- I (<1%) is biologically active [17]. IGFBP-1, also produced in the liver, modulates the transport of IGF-I from plasma to tissues, thereby increasing its bioavailability [18]. Circulating IGFBP-1 levels are inversely regulated by insulin at transcription level; consequently, insulin deficiency in T1DM is associated with elevated IGFBP-1 levels [19,20]. These high IGFBP-1 concentrations are linked to late diabetes complications [21,22].

Patients with T1DM typically exhibit a distinct endocrine profile characterized by reduced serum IGF-I, increased growth hormone (GH), and elevated IGFBP-1, largely due to low hepatic insulin concentration [23,24]. This state suggests hepatic GH resistance, which further contributes to decreased IGF-I synthesis [25]. As IGF-I plays essential roles in cell survival, proliferation, and pancreatic beta-cell protection [26], this dysregulation may have significant pathophysiological consequences. Furthermore, elevated IGFBP-1 not only reduces IGF-I bioavailability but may also exert IGF-independent effects through integrin receptor binding [27,28].

While the individual roles of oxidative stress and IGF-system dysregulation in T1DM are recognized, their potential interplay in young adults remains poorly characterized. Therefore, this study aimed to analyze plasma concentrations of key antioxidant (CoQ10, vitamin E, Grx activity) and oxidative stress (ox-LDL) biomarkers, alongside components of the IGF system (IGF-I, IGFBP-1) in young adults with T1DM compared to age-matched healthy controls. The selection of these biomarkers is supported by previous research indicating that lifestyle and nutritional factors can modulate both the IGF-system and antioxidant status [29], and that supplementation with antioxidants like CoQ10 can influence IGF-levels [30]. A further aim was to investigate potential correlations between these oxidative and IGF-system biomarkers to elucidate a possible mechanistic link in the early pathogenesis of cardiovascular disease in this population.

## 2. Materials and Methods

### 2.1. Study Subjects

This study included 129 patients with type 1 diabetes mellitus (T1DM) (76 females, 53 males; mean age 26.97 ± 0.7 years) and 95 healthy controls (61 females, 34 males; mean age 27.35 ± 0.68 years), all recruited from Brazil. The patients had early-onset T1DM, diagnosed in childhood or early adulthood. There was no significant age difference between the groups (*p* = 0.72). Control participants self-reported good health had no history of major disease and were not taking any regular medications. The study protocol was approved by the Brazilian Ethics Committee. Fasting venous blood samples (10 mL) were collected into EDTA-containing tubes, centrifuged at 2000× *g* for 10 min at 4 °C, and the resulting plasma was aliquoted and stored at −20 °C prior to shipment on dry ice for analysis.

### 2.2. HPLC Analysis of CoQ10 and Vitamin E

Coenzyme Q10 (CoQ10) and vitamin E (α-tocopherol) were extracted from plasma using a modified protocol described elsewhere. Briefly, plasma was mixed with a CoQ6 internal standard, methanol, and hexane. The upper hexane phase was collected, and the extraction was repeated with fresh hexane. The combined hexane fractions were evaporated until dry under a stream of nitrogen gas. The dried extracts were stored at −20 °C until analysis. Prior to injection, samples were reconstituted in chloroform:methanol (2:1, *v*/*v*). Analysis was performed using a Shimadzu LC-10AD high-performance liquid chromatography (HPLC) system (Shimadzu Corporation, Kyoto, Japan) equipped with a Hypersil ODS reversed-phase column (Thermo Fisher Scientific, MA, USA). A linear gradient was applied from methanol/water (90:10, *v*/*v*) to methanol/2-propanol/hexane (40:20:40, *v*/*v*/*v*) over 30 min at a flow rate of 1.5 mL/min. Detection was performed by monitoring absorbance at 275 nm (for CoQ10) and 292 nm (for vitamin E).

### 2.3. Analysis of Lipids and Oxidative Stress Biomarkers

Plasma total cholesterol was measured enzymatically using a standard spectrophotometric method [31]. Apolipoprotein A (ApoA) and Apolipoprotein B (ApoB) levels were determined as previously described [32]. High-density lipoprotein (HDL), low-density lipoprotein (LDL), and triglyceride levels were assessed using standardized clinical laboratory methods [4].

Oxidized LDL (ox-LDL) was quantified using a commercial enzyme-linked immunosorbent assay (ELISA) kit (Mercodia AB, Uppsala, Sweden) according to the manufacturer’s instructions.

Glutaredoxin (Grx) activity was measured using a commercial fluorescent assay kit (IMCO Corporation Ltd., Stockholm, Sweden). Plasma (20 µL) was incubated with a fluorescently labelled bovine serum albumin (BSA) substrate, and the increase in fluorescence emission at 540 nm (excitation at 340 nm) was recorded over time using a VICTOR3 multilabel plate reader (PerkinElmer, Waltham, MA, USA). Recombinant human Grx1 was used as the standard [33].

### 2.4. IGF-I and IFBP-1 Analysis

Total insulin-like growth factor-I (IGF-I) was analyzed using an in-house radioimmunoassay (RIA) after sample pre-treatment with acid-ethanol to dissociate IGF-binding proteins (IGBPs), followed by cryoprecipitation to remove residual binding proteins. A des (1–3) IGF-I tracer was used to minimize interference from any remaining IGFBPs [34]. IGF-I levels were expressed as standard deviation (SD) scores based on age- and sex-adjusted Swedish reference values (*n* = 122, age range 20–60 years).

Insulin-like growth factor binding protein-1 (IGFBP-1) was quantified using a specific in-house RIA [35]. For the assay, 50 µL of plasma was used per tube. The intra-assay and inter-assay coefficients of variation (CV) for the IGF-I and IGFBP-1 assays were <5% and <10%, respectively.

### 2.5. Statistical Analysis

Data is presented as mean ± SD unless otherwise stated. Differences between the T1DM and control groups were assessed using Student’s *t*-test for continuous variables. Associations between variables were evaluated using Spearman’s rank correlation coefficient. A two-tailed *p*-value of <0.05 was considered statistically significant. All analyses were performed using GraphPad Prism version 10.

## 3. Results

### 3.1. Clinical Characteristics of Participants

The baseline demographic and clinical characteristics of participants are presented in Table 1. The two groups were comparable regarding age and sex distribution. The mean age was 26.97 ± 0.70 years in the type 1 diabetes group and 27.35 ± 0.68 years among controls (*p* = 0.72). Females represented 58.9% of the diabetes group and 64.2% of the control group (*p* = 0.42). The average duration of diabetes was 11.24 ± 6.67 years. As expected, individuals with type 1 diabetes had significantly higher HbA1c levels (9.18 ± 0.18%) compared with controls (5.20 ± 0.04%, *p* < 0.0001).

### 3.2. Antioxidant Status and Oxidative Stress Markers Glutaredoxin Activity and Oxidized LDL

Glutaredoxin (Grx) activity was significantly higher in patients with T1DM than in healthy age matched controls (*p* < 0.0001). Similarly, plasma oxidized LDL (ox-LDL) levels were significantly elevated in the T1DM group (*p* = 0.0002) (Table 2).

### 3.3. Coenzyme Q10 and Vitamin E

Plasma coenzyme Q10 (CoQ10) levels were elevated in both groups. The mean CoQ10 level was higher in the T1DM group, but this difference was not statistically significant. Vitamin E levels were slightly lower in the T1DM group, with considerable interindividual variation observed in both cohorts (Table 3).

### 3.4. Lipid and Glucose Profiles

The lipid and metabolic profiles of the study participants are summarized in Table 2.

#### 3.4.1. HDL and LDL

High-density lipoprotein (HDL) cholesterol was significantly higher in the T1DM group (1.278 ± 0.046 mmol/L) compared to the control group (0.933 ± 0.035 mmol/L; *p* < 0.0001). Low-density lipoprotein (LDL) cholesterol levels did not differ significantly between groups (T1DM: 1.95 ± 0.11 mmol/L vs. Control: 1.85 ± 0.10 mmol/L; *p* = 0.478). Consequently, the LDL/HDL ratio was significantly lower in the T1DM group (1.52 ± 0.07) than in the control group (1.98 ± 0.07; *p* < 0.0001).

#### 3.4.2. Cholesterol and Triglycerides

Total cholesterol was significantly higher in the T1DM group (3.89 ± 0.16 mmol/L) than in controls (3.41 ± 0.13 mmol/L; *p* = 0.021). Triglyceride levels showed no significant difference between the groups.

#### 3.4.3. Apolipoproteins

Apolipoprotein A1 (ApoA1) levels were similar across groups. In contrast, apolipoprotein B (ApoB) levels and the ApoB/ApoA1 ratio were both significantly elevated in the T1DM group (*p* = 0.0103 and *p* = 0.0316, respectively), indicating a pro-atherogenic lipid profile and increased cardiovascular risk.

#### 3.4.4. Glycemic Control

As expected, glycated hemoglobin (HbA1c) was significantly elevated in the T1DM group (9.18 ± 0.18%; 77.0 ± 2.0 mmol/mol) compared to controls (5.20 ± 0.035%; 33.3 ± 0.4 mmol/mol; *p* < 0.0001). No significant age difference was observed between the groups.

### 3.5. IGF-I and IGFBP-1 Concentrations

**Figure 1:**
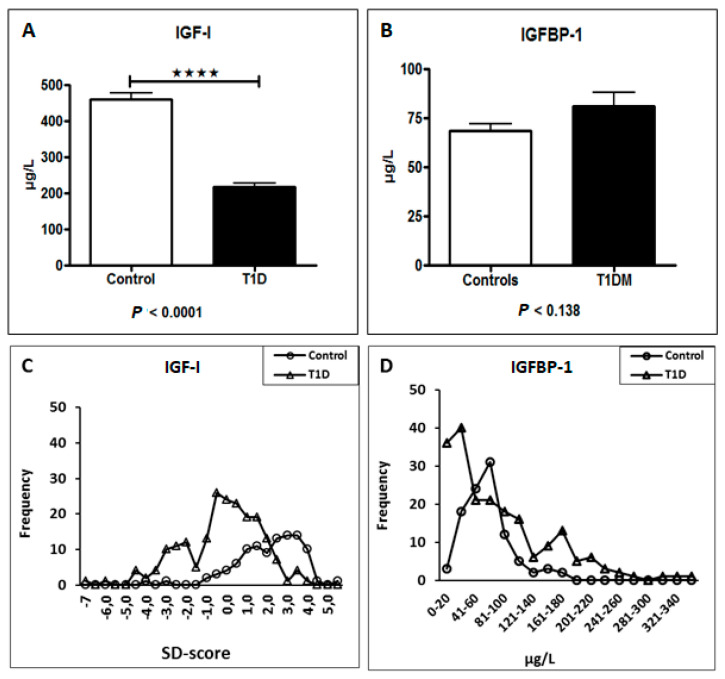
Alterations in the IGF-system in young adults with Type 1 Diabetes. (**A**) Plasma IGF-I levels were significantly lower in the T1DM group compared to healthy controls (**** *p* < 0.0001). (**B**) IGFBP-1 levels were higher in the T1DM group, but the difference was not statistically significant (*p* = 0.138). (**C**) Age-adjusted IGF-I standard deviation (SD) scores were significantly reduced in patients with T1DM. (**D**) Frequency distribution of IGFBP-1 levels in the T1DM group shows a bimodal distribution, suggesting the presence of patient subgroups. Data are presented as mean ± SD (**A**,**B**) or box plots showing median, quartiles, and range (**C**). Statistical significance was determined by Student’s *t*-test.

Plasma insulin-like growth factor-I (IGF-I) was significantly lower in patients with T1DM than in controls (*p* < 0.0001) (Figure 1A). Insulin-like growth factor binding protein-1 (IGFBP-1) levels were higher in the T1DM group, but this difference did not reach statistical significance (*p* = 0.138) (Figure 1B). Age-adjusted IGF-I standard deviation (SD) scores were substantially lower in the T1DM cohort Figure 1C. The distribution of IGFBP-1 levels within the T1DM group indicated the presence of subgroups with elevated concentrations Figure 1D.

**Figure 2:**
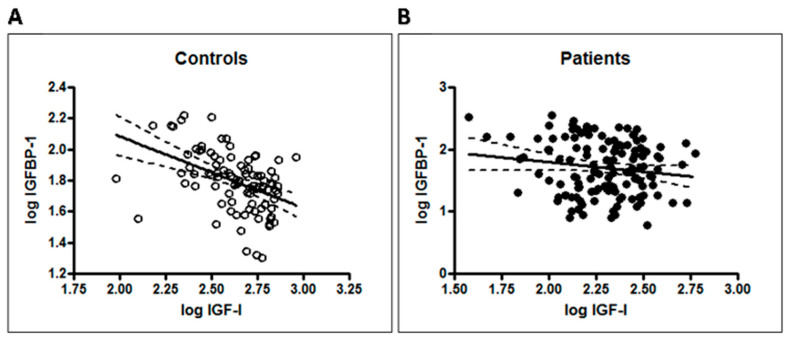
Disruption of the physiological correlation between IGF-I and IGFBP-1 in Type 1 Diabetes. (**A**) A significant negative correlation was observed between log-transformed IGF-I and IGFBP-1 levels in the healthy control group (r = −0.45, *p* < 0.0001). (**B**) This significant correlation was absent in the T1DM group (r = −0.14, *p* = 0.08). Solid lines represent the regression line; shaded areas represent the 95% confidence interval.

A significant negative correlation between IGF-I and IGFBP-1 was observed in the control group (r = −0.45, r^2^ = 0.20, *p* < 0.0001) (Figure 2A). This correlation was absent in the T1DM group (r = −0.14, r^2^ = 0.02, *p* = 0.08) (Figure 2B).

## 4. Discussion

This study analyzed cardiovascular risk factors, focusing on the IGF-system and plasma antioxidant levels, in a cohort of young Brazilian adults with type 1 diabetes (T1DM) compared to age-matched healthy controls. The key findings include significantly elevated oxidative stress biomarkers (ox-LDL, Grx activity), a pro-atherogenic lipid profile (elevated ApoB and ApoB/ApoA1 ratio, increased HDL), and a disrupted IGF-axis (significantly lower IGF-I with slightly higher IGFBP1 but no correlation between IGF-I and IGFBP-1) in patients with T1DM, despite evidence of compensatory antioxidant upregulation.

Furthermore, our finding of a significantly elevated ApoB/ApoA1 ratio in young adults with T1DM provides critical insight into their cardiovascular risk profile. This ratio is recognized as a more powerful predictor of cardiovascular events than conventional lipid measures, as it reflects the fundamental balance between the total number of atherogenic, cholesterol-enriched particles (ApoB) and the primary component of anti-atherogenic HDL (ApoA1) [36,37]. The elevated ratio observed here indicates a pronounced pro-atherogenic state, capturing the essence of diabetic dyslipidemia that persists even in this young cohort.

The chronic hyperglycemic state characteristic of T1DM promotes oxidative stress, which contributes to enhanced lipid peroxidation. The oxidation of low-density lipoprotein (LDL) is a critical initiating factor in the development of atherosclerosis. Consistent with this, our study found that levels of oxidized LDL (ox-LDL) were approximately 1.5 times higher in patients with T1DM than in healthy controls. This aligns with evidence suggesting that lipid peroxidation, a key early event in atherosclerosis, can begin in childhood in individuals with T1DM [38,39].

Contrary to the elevated cardiovascular risk, high-density lipoprotein (HDL) cholesterol levels are often reported to be normal or elevated in T1DM, a finding confirmed in this work. One proposed explanation is the increased concentration of adiponectin in these patients, which may reflect a compensatory mechanism due to its anti-inflammatory and antiatherogenic properties [40]. However, it is also possible that elevated adiponectin levels reflect a state of adiponectin resistance, potentially due to the downregulation of its receptors [41].

Our results showed significantly impaired glycemic control in the T1DM group (HbA1c 9.18 ± 0.18%) compared to controls (5.20 ± 0.035%). A previous study in youth with T1DM found that individuals with suboptimal glycemic control exhibited elevated lipid levels, whereas those with optimal control had profiles comparable to non-diabetic controls [42,43]. The longer average disease duration in our cohort (11.24 ± 6.67 years) is likely to exacerbate cardiovascular risk in this young population, particularly among those with persistent hyperglycemia.

The observed upregulation of biomarkers like Grx activity indicates an adaptive cellular response to oxidative stress. However, this compensation appears insufficient to fully counteract oxidative damage, as evidenced by significantly elevated ox-LDL levels. This suggests that both chronic hyperglycemia and insulin deficiency continue to drive cardiovascular risk pathways despite these intrinsic compensatory mechanisms. The findings of our previous intervention study, where CoQ10 supplementation improved oxidative status in diabetics, support the potential for targeted antioxidant therapy to augment these natural defenses [30]. However, the literature presents conflicting results, with some studies questioning the oxidative stress hypothesis in T1DM despite evidence of reduced total antioxidant defense [44], highlighting the need for further research.

We also identified a significantly altered IGF-axis. IGF-I levels were approximately 2.5-fold lower in our T1DM group, consistent with previous reports attributing this reduction to insulinopenia and hepatic GH resistance [25]. A large longitudinal study similarly identified poor metabolic control and longer disease duration as key determinants of reduced IGF-I levels, underscoring the importance of insulin availability for maintaining normal IGF-I concentrations. We also found elevated IGFBP-1 levels in the T1DM group, which prior research suggests may be due to decreased DNA methylation of the IGFBP-1 gene promoter leading to higher expression [45]. While the elevation of IGFBP-1 in our T1DM cohort was not statistically significant, the direction of this change is consistent with the established literature describing elevated IGFBP-1 in T1DM due to insulin deficiency. The more critical finding, however, is the functional dysregulation revealed by the lost correlation with IGF-I [18]. Crucially, we observed a significant negative correlation between IGF-I and IGFBP-1 in healthy controls, a regulatory relationship that was absent in the T1DM group. This disrupted interplay, first described by Brismar’s group and confirmed in our cohort, suggests a profound dysregulation of the IGF system in T1DM that may contribute to metabolic and vascular complications [21].

### Limitations

This study has several limitations. Its cross-sectional design allows for the identification of associations but not the determination of causality. The sample was recruited from a single region in Brazil, which may limit the generalizability of the findings to other ethnic and geographic populations. While we measured key biomarkers, the absence of data on adiponectin levels or direct measures of insulin resistance (e.g., HOMA-IR) prevents a deeper mechanistic exploration of the observed lipid profile and IGF-system dysregulation. Furthermore, the considerable interindividual variation in some biomarkers, such as vitamin E, may have limited our power to detect significant differences. Most importantly, the lack of anthropometric data, such as body mass index (BMI) and waist circumference, is a limitation, as these factors are known to influence both oxidative stress and the IGF-system. The inability to adjust for these potential confounders should be considered when interpreting the results.

## 5. Conclusions

In summary, our study reinforces the association between oxidative stress, dyslipidemia, and a disrupted IGF-system in young adults with T1DM. The loss of the physiological correlation between IGF-I and IGFBP-1 presents a novel insight into the metabolic dysregulation inherent to this disease. These findings suggest that the early pathophysiological processes of cardiovascular disease are already active in young adults with T1DM. Therefore, early detection and targeted management of these biomarkers—particularly aiming for optimal glycemic control—may be crucial for preventing long-term vascular complications in this vulnerable population. Future longitudinal studies are needed to confirm these associations and explore therapeutic interventions aimed at modulating oxidative stress and the IGF-axis.

## Figures and Tables

**Table 1:** Baseline Characteristics of the Study Population.

Characteristic	T1DM Group (*n* = 129)	Control Group (*n* = 95)	*p*-Value
Age (years)	26.97 ± 0.70	27.35 ± 0.68	*p* < 0.721
Sex (% Female)	76 (58.9%)	61 (64.2%)	*p* < 0.42
Diabetes duration (years)	11.24 ± 6.67	-	-
HbA1c (%)	9.18 ± 0.18	5.20 ± 0.035	*p* < 0.0001

**Table 2:** Antioxidant Status and Oxidative Stress Markers.

	T1DM Group (*n* = 129)	Control Group (*n* = 95)	*p*-Value
Grx Activity (ng/mL)	13.21 ± 0.66	7.59 ± 0.45	*p* < 0.0001
ox-LDL (mU/L)	29.9 ± 7.26	18.82 ± 4.46	*p* < 0.0002
Coenzyme Q10 (µmol/L)	2.12 ± 0.25	1.67 ± 0.16	*p* < 0.139
Vitamin E (µmol/L)	55.28 ± 9.38	69.0 ± 13.33	*p* < 0.404

**Table 3:** Lipid and metabolic profiles.

	T1DM Group	Control Group	*p*-Value
Glycaemic Control	(*n* = 129)	(*n* = 95)	
HbA1c (%)	9.18 ± 0.18	5.20 ± 0.035	*p* < 0.0001
Lipid Profile			
Total Cholesterol (mmol/L)	3.89 ± 0.16	3.41 ± 0.13	*p* < 0.0209
HDL (mmol/L)	1.28 ± 0.046	0.93 ± 0.035	*p* < 0.0001
LDL (mmol/L)	1.95 ± 0.11	1.85 ± 0.10	*p* < 0.4784
Triglycerides (mmol/L)	1.40 ± 0.072	1.43 ± 0.14	*p* < 0.8408
Atherogenic Indices			
LDL/HDL ratio	1.52 ± 0.07	1.98 ± 0.072	*p* < 0.0001
ApoB/ApoA1 ratio	0.46 ± 0.018	0.53 ± 0.027	*p* < 0.0316
Apolipoproteins			
ApoA1 (g/L)	1.74 ± 0.06	1.72 ± 0.07	*p* < 0.8373
ApoB (g/L)	0.91 ± 0.042	0.77 ± 0.032	*p* < 0.0103

## Data Availability

The original contributions presented in this study are included in the article. Further inquiries can be directed to the corresponding author.

## Abbreviations

ApoA: Apolipoprotein A; ApoB: Apolipoprotein B; CoQ10: Coenzyme Q10; Grx: Glutaredoxin; GH: Growth hormone; HDL: High-density lipoprotein; IGF: Insulin-like growth factor; IGFBP-1: Insulin-like growth factor binding protein-1; LDL: Low-density lipoprotein; OX-LDL: Oxidized low-density lipoprotein; ROS: Reactive oxygen species; T1DM: Type-1 diabetes mellitus.
